# Transcriptomics Reveal Several Novel Viruses from Canegrubs (Coleoptera: Scarabaeidae) in Central Queensland, Australia

**DOI:** 10.3390/v14030649

**Published:** 2022-03-21

**Authors:** Kayvan Etebari, Pauline Lenancker, Kevin S. Powell, Michael J. Furlong

**Affiliations:** 1 School of Biological Sciences, The University of Queensland, St. Lucia, QLD 4072, Australia; m.furlong@uq.edu.au; 2 Sugar Research Australia, 71378 Bruce Highway, Gordonvale, QLD 4865, Australia; plenancker@sugarresearch.com.au (P.L.); kpowell@sugarresearch.com.au (K.S.P.)

**Keywords:** viriome, beetles, insect virus discovery, RNA viruses, sugarcane

## Abstract

Canegrubs (Coleoptera: Scarabaeidae) are major pests of sugarcane crops in Australia, but despite long-term and intensive research, no commercially viable biological control agents have been identified. We used the RNA-Seq approach to explore the viriomes of three different species of canegrubs from central Queensland, Australia to identify potential candidates for biological control. We identified six novel RNA viruses, characterized their genomes, and inferred their evolutionary relationships with other closely related viruses. These novel viruses showed similarity to other known members from picornaviruses, benyviruses, sobemoviruses, totiviruses, and reoviruses. The abundance of viral reads varied in these libraries; for example, *Dermolepida albohirtum* picorna-like virus (9696 nt) was built from 83,894 assembled reads while only 1350 reads mapped to *Lepidiota negatoria* beny-like virus (6371 nt). Future studies are essential to determine their natural incidence in different life stages of the host, biodiversity, geographical distributions, and potential as biological control agents for these important pests of sugarcane.

## 1. Introduction

Sugarcane is grown in tropical and subtropical regions across the globe, and over 1500 insect species have been reported to feed on sugarcane plants [1]; estimates suggest that 20–40% of global industry losses are due to pests and diseases [2]. In Australia, canegrubs (Coleoptera: Scarabaeidae) are major insect pests of sugarcane crops, and they cause significant yield losses in some growing regions [2,3]. The Australian canegrubs constitute a complex of endemic melolonthine scarab larvae. There are 119 recognised species of Melolonthini in Australia [4], of which 75 are found in Queensland. Nineteen species of these beetles, from the genera *Lepidiota* (ten species), *Antitrogus* (four species), *Alepida* (three species), *Dermolepida* (one species), and *Rhopaea* (one species) [2,4] are recognised as sugarcane pests, and of these, the greyback (*Dermolepida albohirtum*), French (*Lepidiota frenchi*), and negatoria canegrubs (*Lepidiota negatoria*) are the most damaging and widespread [2]. While the greyback canegrub is the most geographically widespread (occurring in five production regions in Australia) and the most economically damaging (root feeding by its larvae can cause significant financial losses to the industry [3]), the negatoria canegrubs are the only important pest in some areas of central and southern Queensland [5].

Despite significant research efforts to find effective biological control agents for cane grubs, including the evaluation of the entomopathogenic fungus *Metarhizium* *anisopliae*, no commercially viable biological control strategy is available. One strain of *M. anisopliae* has been formulated and was commercialized as BioCane^TM^ [5,6]. It was only recommended for application where the risk of pest pressure is medium-low [5,6], but it has been subsequently discontinued from the market.

Currently, the Australian sugarcane industry relies solely on the neonicotinoid insecticide imidacloprid for the control of canegrubs. This approach is not sustainable as overreliance on a single compound presents a significant risk that populations will ultimately evolve resistance [2]. Furthermore, these compounds have considerable nontarget effects [7] that have led to them being banned for agricultural use in Europe. In Australia, high levels of imidacloprid residues have been detected on sugarcane farms and in the wider environment (waterways and rivers). Furthermore, there are growing concerns regarding the common detection of imidacloprid residues in the Great Barrier Reef catchment and marine environment [7,8]. Due to these concerns, the Australian Pesticides and Veterinary Medicines Authority is currently reviewing the use of imidacloprid and could ultimately limit its use in Australian sugarcane, restricting the only current management option for canegrubs.

Previous studies have shown that canegrub larvae are susceptible to a number of pathogens that could, in some cases, be responsible for significant suppression of their local populations. The protozoan *Adelina* sp., the fungus *M. anisopliae*, and the bacterium *Paenibacillus popilliae* are the major known pathogens of canegrubs in Australia [5]. Sallam et al. (2011) reported high levels of larval mortality in canegrubs from far north Queensland, but the cause of this mortality was not determined [9]. An unidentified entomopoxvirus was also reported from greyback canegrub larvae in 1975; however, available technologies at the time did not allow the researchers to investigate the incidence of the virus in different canegrub populations [10].

Several viral pathogens have been reported from scarab beetles across the world (Table 1), and some of these can induce significant mortality in pest populations [11,12,13,14,15,16,17,18,19,20,21,22,23,24,25,26,27,28,29,30,31,32]. The effective control of the coconut rhinoceros beetle, *Oryctes rhinoceros* (Coleoptera: Scarabaeidae), in southeast Asia and the South Pacific islands by a double stranded DNA virus (OrNV: Oryctes rhinoceros nudivirus) [25] is widely considered to be a landmark example of classical biological control, and it represents one of the few examples of successful classical biological control involving an entomopathogen [33]. OrNV can infect and replicate in other coleopteran cell lines, including DSIR-HA-1179 (an embryonic cell line originally derived from the black African beetle, *Heteronychus arator* [34], which is a pest of sugarcane and other crops) and FRI-AnCu-35 (an embryonic cell line of the cupreous chafer, *Anomala cuprea* (Coleoptera: Scarabaeidae)) [35]. Currently, very few insect nudiviruses have been described [36], but members of the family *Nudiviridae* have great potential for use as biocontrol agents of insect pests, including canegrubs.

Identifying viral pathogens is the first step in investigating their potential as biocontrol agents for canegrubs and other beetle pests. In this study, we have sequenced several RNA viruses from three canegrub species for the first time and conducted phylogenetic analyses to identify novel insect-specific viruses, characterize their genomes, and infer their evolutionary relationships with other closely related viruses. We focused our work on three of the main canegrub species that attack sugarcane in Australia: *D. alborhirtum*, *L. frenchi*, and *L*. *negatoria* [2,5]. Here, we only report the viruses for which putative complete genome sequences have been identified; several short viral fragments that may be endogenous viral elements (EVEs) were excluded from this study.

## 2. Materials and Methods

### 2.1. Insect Collection and RNA Extraction

Adult beetles of three different species, *D. albohirtum* (*n* = 15), *L.* *frenchi* (*n* = 10), and *L. negatoria* (*n* = 15), were collected from commercial sugarcane farms in Mackay, Central Queensland, Australia in November 2020. Adult beetles were transported to the University of Queensland for viral discovery based on next-generation sequencing.

After 72 h of induced starvation, their gut tissue was dissected and preserved in an RNA protect reagent for RNA extraction and further analysis. The total RNA was extracted from 250 μL of insect gut tissue homogenates using Trizol following the manufacturer’s protocol (Life Technologies, Rockville, MD, USA). The RNA samples were treated with DNase I for 1 h at 37 °C, and then, their concentrations were measured using a spectrophotometer; integrity was ensured through analysis of RNA on a 1% (*w*/*v*) agarose gel. After checking the RNA quality, total RNA samples were submitted to the Genewiz sequencing facility (Suzhou, China) for library preparation and strand-specific total RNA and mRNA (poly(A)-enriched) sequencing on the NovaSeq platform. For some samples from each species that were used in total RNA sequencing, the eukaryote ribosomal RNA was removed using a VAHTS Total RNA-seq (HMR) Library Prep Kit for Illumina.

### 2.2. RNA-Seq Data Analysis

**Figure 1:**
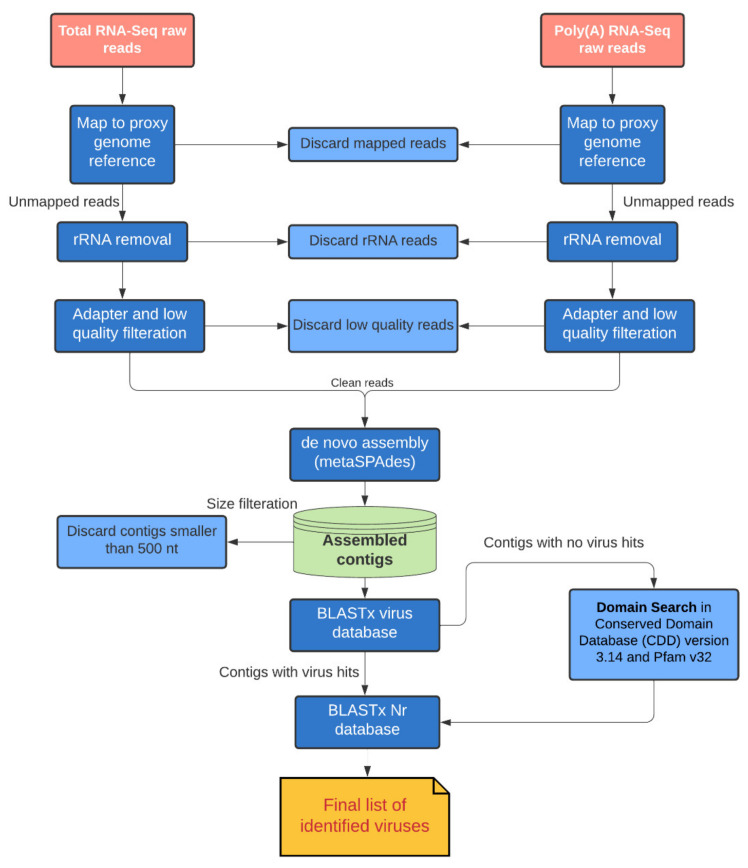
The pipeline for virus discovery in canegrubs (Coleoptera: Scarabaeidae).

The CLC genomics workbench 21.0.5 and OmicsBox 2.0.36 were used to process the data, and our virus discovery pipeline is summarized in Figure 1. Libraries were trimmed from any vector or adapter sequences remaining. Low quality reads (quality score below 0.05) and reads with more than two ambiguous nucleotides were discarded. In the absence of a reference genome, we mapped the trimmed reads to the genomes of two other Scarabaeidae beetles, *Oryctes rhinoceros* (GCA_020654165.1) and *Onthophagus taurus* (GCF_000648695.1), as proxy genome references to discard insect-specific reads. We applied a minimum length fraction = 0.5, similarity fraction = 0.8, maximum mismatches = 2, insertion cost = 3, and deletion cost = 3 as mapping criteria in CLC Genomics Workbench. The unmapped reads were retained for downstream analysis after removing rRNA with SortMeRNA [37]. We used metaSPAdes [38] with automatic k-mer size (default parameters) for de novo assembly in the metagenomic mode.

BLASTx was used to identify sequence similarity of all assembled contigs with the protein database (nr). To detect highly divergent viruses, we also performed domain-based searches by comparing the assembled contigs against the Conserved Domain Database (CDD) version 3.14 and Pfam v32, with an expected value threshold of 1 × 10^−3^. Sequences with positive hits to virus polymerase (RNA-dependent RNA polymerase (RdRP) domain: cd01699) were retained. Any sequences with a positive hit were checked with BLASTn against the nucleotide database (nt) to remove any potential false positive outcomes.

Contig sequences with a high degree of similarity to viral proteins were then checked for complete ORFs. ORFs with a minimum length of 150 aa were detected in the CLC Genomic Workbench by using the standard genetic code. We used this tool for viral genome annotation such as the identification of a signal peptide, potential glycosylation sites, and transmembrane domains. We also remapped the RNA-Seq data to putative virus sequences to inspect for sufficient coverage and possible misassembly. The CLC Genomic Workbench’s RNA-Seq function (min. length fraction = 0.9, maximum mismatches = 2, insertion cost = 3, deletion cost = 3) on a nonstrand-specific option was used.

### 2.3. Phylogenetic Analysis

The deduced amino acid sequence of predicted ORF regions or RdRP of newly identified viruses were used to calculate their phylogenetic relationship with other members of each respective family. Closely related viruses from the BLASTp analysis of the NCBI nonredundant protein database were downloaded. Multiple amino acid sequence alignments with relevant reference sequences were performed with MUSCLE. We used ModelFinder (http://www.iqtree.org, accessed on 30 January 2022) to check the best-fit model out of 546 protein models. The maximum likelihood phylogenetic trees were inferred using a JTT substitution matrix and assuming a discretised gamma rate distribution with four rate categories and with 1000 bootstraps. The shape parameter of the gamma distribution was fixed at 1.0 in the CLC Genomics Workbench.

## 3. Results and Discussion

As gut tissue is the most active site for viral entry and replication in other scarabs, we performed Illumina-based high-throughput sequencing on poly(A)-enriched and total RNAs extracted from gut tissues of three canegrubs collected from Queensland, Australia. In total, 256,291,462 and 218,995,416 raw reads were produced from three total RNA libraries and three poly(A)-enriched RNA libraries, respectively. The number of unmapped reads to proxy genomes and the assembled contigs and scaffolds are summarized in Table 2. Deep sequencing raw data were deposited in the National Centre for Biotechnology Information’s (NCBI’s) Sequence Read Archive (SRA) and are accessible through BioProject series accession number PRJNA798832. In this study, we identified six novel RNA viruses, characterized their genomes, and inferred their evolutionary relationships with other closely related viruses.

### 3.1. Dermolepida Albohirtum Picorna-like Virus (DaPV1)

**Figure 2:**
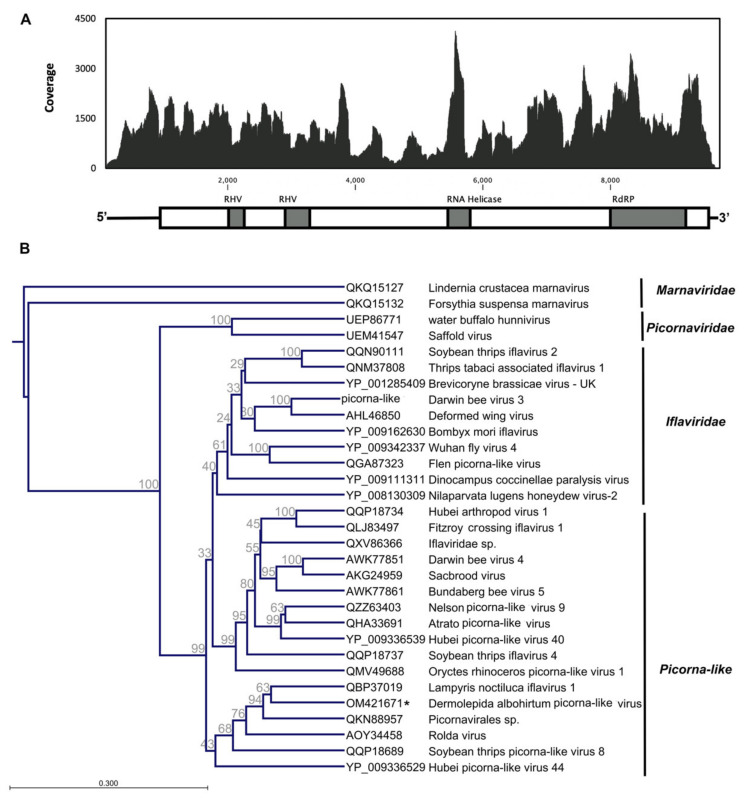
Schematic diagram of the genome organization and coverage of Dermolepida albohirtum picorna-like virus (**A**). The maximum likelihood phylogeny of the novel picorna-like virus (DaPV1) and previously reported viruses in the *Picornavirales* based on deduced amino acid sequence of conserved RdRP domain. DaPV1 is clustered with other members of unclassified *Picornavirales* and some members of family *Iflaviridae*. We used two members of family *Marnaviridae* as outgroups in this analysis. The identified virus in this study is shown with an asterisk (**B**).

A novel picorna-like virus, tentatively named dermolepida albohirtum picorna-like virus (DaPV1), was identified from the greyback canegrub (*D. albohirtum*) samples, which were collected in Mackay, Central Queensland, Australia. The genome included a single open reading frame encoding a 2909 product (Figure 2A). Conserved structural domains, such as the picornavirus capsid protein, RNA helicase, and RdRp, were identified through the Pfam domain search. The deduced amino acid sequence of identified open reading frame shared a 43.5 and 33.6% sequence identity with Apis mellifera iflavirus 2 and Lampyris noctiluca iflavirus 1, respectively. The 5′ untranslated region (5′UTR) and the short 3′ UTR contained 833 and 136 nucleotides, respectively. We identified this virus in the sample that was sequenced by total RNA-Seq without the poly(A) enrichment step, and we could not detect a naturally occurring poly(A) tail for this novel virus. The viral genomic sequence was found to be AU-rich (63.7%), and the viral polyprotein sequence has a predicted molecular mass of 325.307 kDa and theoretical isoelectric point (pI) of 7.15. Three amino acids of serine (S), leucine (L), and valine (V) have the highest frequencies in the predicted polyprotein sequence. The deduced amino acid sequence contains 1371 hydrophobic and 927 hydrophilic residues.

The DaPV1 genome was built from 83,894 assembled reads that showed an average coverage of 1275×. Maximum coverage (4129×) was detected towards the RNA helicase and RdRp conserved domain regions. This high coverage suggests that this virus might actively replicate in the host. The annotated genomic sequence of this virus has been deposited in GenBank under the accession number OM421671.

The maximum likelihood phylogeny separated this novel virus (DaPV1) from other members of the *Picornaviridae* and *Marnaviridae* but indicate that it shares common ancestry with the iflaviruses as well as other picorna-like viruses. Although it shows a high degree of similarity with other putative iflaviruses, the phylogenetic analysis clustered this novel virus with other unclassified picorna-like viruses that have previously been reported from insects (Figure 2B). In the past few years, several isometric virions have been assigned under the supergroup “picorna-like viruses”, and they have been reported from a wide range of insects including Coleoptera, for example *Oryctes rhinoceros* (Scarabaeidae) [27], *Aulacophora lewisii* (Chrysomelidae) [39], *Harmonia axyridis* (Coccinellidae) [40], and *Cheilomenes sexmaculata* (Coccinellidae) [41]. 

Recently, several *Picornavirales* and other insect-specific viruses have been reported from birds and bats, and the insectivorous feeding of these vertebrates might be the cause of this spillover and cross-species transmission [42]. DaPV1 shows 41.6% similarity with 70% coverage to a picorna-like viruses that have been isolated from brown shrike (*Lanius cristatus*) in China. Additionally, another virus found in the vesper bat (*Yuma myotis*) from Washington showed 38.4% similarity with 53% coverage to the greyback canegrub picorna-like virus (DaPV1). Further investigations are required to determine if these insect viruses can replicate and infect birds, bats, and other vertebrates.

There is a growing body of evidence showing that iflaviruses exclusively infect arthropods, but in most cases, their infections remain unnoticed, and the majority of infections are asymptomatic while only small portions of infected hosts produce severe symptoms [43]. However, the recent discoveries through deep sequencing approaches make them an ever-growing group of viruses. Some iflaviruses and other picorna-like viruses can cause clear signs of disease in their hosts (e.g., silkworm and honey bees) while others increase host susceptibility to other pathogens; however, we know little about their diversity and virulence in other insects [44]. It has been shown that coinfection with iflaviruses increases the insecticidal properties of Spodoptera exigua multiple nucleopolyhedrovirus (SemNPV) in beet armyworm larvae [45,46]. These insect-specific small viruses could become important for pest management, but to date, far more attention has been devoted to the arthropod-specific DNA baculoviruses. 

### 3.2. Dermolepida Albohirtum Toti-like Virus (DaTV1)

**Figure 3:**
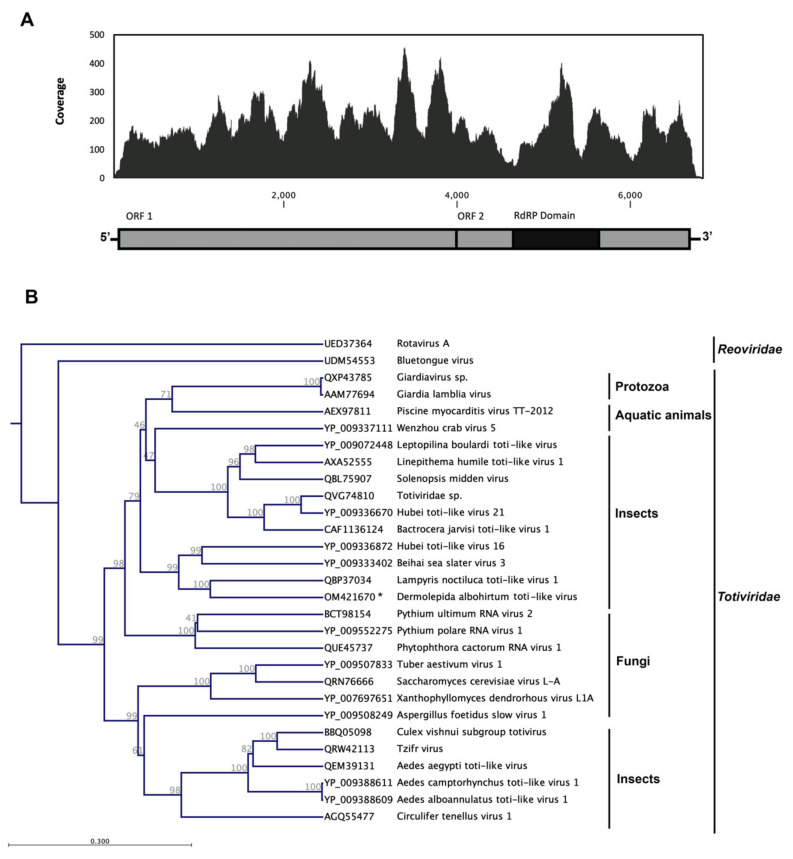
Schematic of the genome organization and coverage of greyback canegrub toti-like virus. The conserved structural domains were identified through a Pfam domain search. RNA-Seq read density plotted along the viral genome. The *Y*-axis shows the number of reads mapped to a particular nucleotide of the virus genome at the *X*-axis (**A**). The maximum likelihood phylogeny of Dermolepida albohirtum toti-like virus (DaTV1) and previously reported viruses in family *Totiviridae* based on deduced amino acid sequence of conserved RdRP domain. We used two members of family *Reoviridae* as outgroups in this analysis. The novel DaTV1is clustered with other members of this family which previously reported from insects and other arthropods. The identified virus in this study is shown with an asterisk (**B**).

Totiviruses are nonenveloped, double-stranded, positive-sense RNA viruses with icosahedral virions, which consist of a single capsid protein [47]. This is the first report of a toti-like virus in Australian greyback canegrubs, which we have tentatively named Dermolepida albohirtum toti-like virus (DaTV1). The complete genome sequence of this novel virus (GenBank accession number: OM421670) consists of a single segment of 6741 bp (Figure 3) with a 38% G + C content. The genome has 69 and 133 nucleotides in its 5′ and 3′ UTR, respectively.

The genome is predicted to encode two large ORFs in which the first ORF is 3984 nt, and encodes a 1328 amino acids (aa) protein that shares 29.4% aa sequence identity (query coverage, 84%) with the hypothetical protein 2 of Hubei toti-like virus 16, which was isolated from spiders in China [48]. This ORF also showed 30.1% aa sequence similarity with 50% query coverage to another toti-like virus (QBP37033.1), which was isolated from adult common glowworm beetles (*Lampyris noctiluca*) in Finland [49]. The Beihai sea slater virus 3 isolated from wharf roaches (*Ligia* sp.) in China had the maximum sequence coverage of 97% (sequence similarity of 24.8%) with the first ORF of this newly identified virus [48]. We could not identify any putative conserved domains or find any similarity with known proteins for this sequence (Figure 3).

The second ORF has one nucleotide overlap with ORF 1 and encodes an 852 aa product with a putative RNA-dependent RNA polymerase (RdRP_4) conserved domain (pfam:02123). It showed the maximum sequence identity (33.8%) and coverage (99%) to Lampyris noctiluca toti-like virus 1 (QBP37034.1). Significant similarity has also been found to other toti-like viruses, which have been identified in terrestrial and aquatic animals, such as the horseshoe crab (Beihai toti-like virus 4), horseflies (Hubei toti-like virus 19), and Penaeid shrimp (Beihai toti-like virus 5). Generally, members of this group have two common ORFs in their genome sequence. We identified two extra small coding regions in 5′ and 3′ of the viral genome. It has been suggested these additional coding sequences potentially produce some proteins that facilitate more complicated infection mechanisms in higher order organisms, with large DNA (genome size) and complex developmental processes, that possess sophisticated immune systems [50]. However, further studies are required to explore the function of these additional ORFs in this group of viruses; this will help our understanding of the evolution of toti-like viruses, their relationships with other members of *Totiviridae*, and how these viruses adapt to infect higher-order hosts.

Traditionally, fungi and protozoa have been recognised as hosts for these viruses, but recent metagenomic analysis has revealed that they also infect higher-order organisms. Several unclassified toti-like viruses have been identified from arthropods, fish, worms, and insectivorous bats [50,51,52]. The disease-causing potential and economic impact of these viruses in agriculture and public health is poorly described.

### 3.3. Dermolepida Albohirtum Reovirus (DaRV)

**Figure 4:**
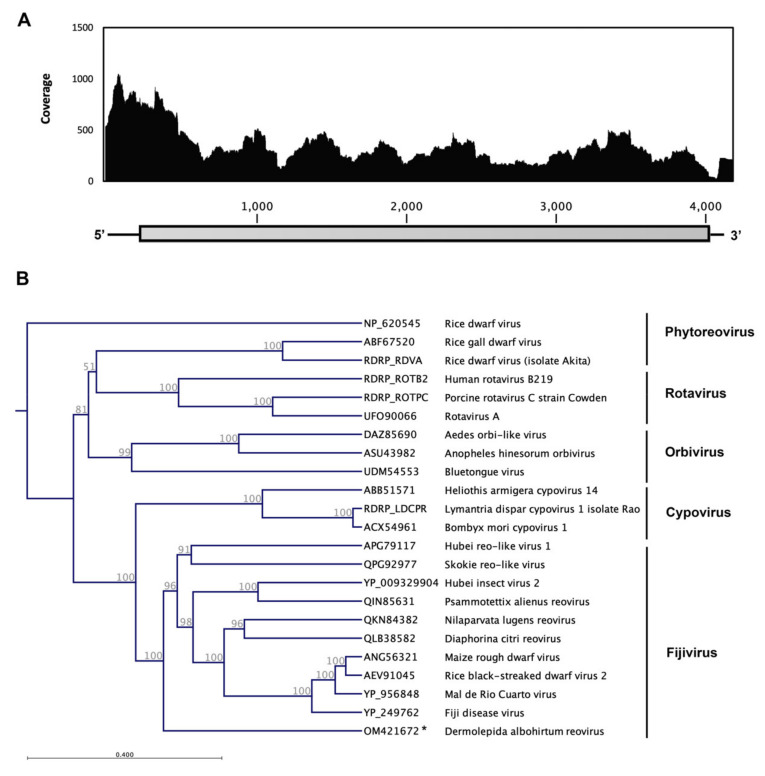
Schematic of the genome organization and coverage of Dermolepida albohirtum reovirus (DaRV). RNA-Seq read density plotted along the viral genome. The *Y*-axis shows the number of reads mapped to a particular nucleotide of the virus genome at the *X*-axis (**A**). The maximum likelihood phylogeny of novel member of the *Reoviridae* family, which identified in *D. albohirtum* (DaRV) and previously reported viruses in the *Reoviridae* family based on deduced amino acid sequence of conserved RdRP domain. The identified virus in this study is shown with an asterisk (**B**).

We identified a single segment genome sequence of a putative virus (GenBank accession number: OM421672) from the *Reoviridae* family and tentatively named it Dermolepida albohirtum reovirus (DaRV). The RNA genome consists of 4122 nucleotides and includes one open reading frame with 1274 aa and the predicted molecular weight of 145.502 kDa with an Isoelectric point 7.32 (Figure 4). The deduced amino acid sequence of its predicted ORF showed 24.9% identity and 75% coverage to RdRP of the Fiji disease virus (YP_249762), which has previously been reported from sugarcane in Australia. It also showed around 25% identity and 70% coverage to RdRp of different isolates of rice black-streaked dwarf virus, which has been isolated across the Asia and Middle East. This viral genome sequence was built by 9099 reads, and it has only been identified in the gut tissue of *D. albohirtum* (Figure 4).

Plant viruses from the family *Reoviridae* (phytoreoviruses, fijiviruses, and oryzaviruses) can replicate in both plants and arthropod vectors, and their main insect vectors are planthoppers and leafhoppers. Sugarcane Fiji disease virus is a type species of *Fijivirus* [53]. Currently, there is no known plant disease associated with this novel virus or any report of Fiji disease-like symptoms in the region. A previous study showed some plant viruses can be found in insect gut cells and that they can enter the hemocoel even through the peritrophic membrane-lined midgut [54]. These canegrubs are root-feeding insects, and they feed on sugarcane roots during their larval stages. The adult beetles are also herbivorous, but they feed on leaves of different trees, such as mango, fig, palms, and, occasionally, sugarcane leaves. They might have acquired this virus when feeding on plant materials or become infected during the larval stage. This novel virus may be an insect-specific virus that only has the same ancestral linkage to plant viruses, or it may correspond to a plant virus (in transit through the gut) and not an insect virus. Further investigation is highly recommended to evaluate its distribution, replication in insect cells, and pathogenicity. Small RNN-Seq analysis can also generate valuable information about possible host RNAi responses to this virus and determine whether the virus is replicating in the host.

*Fijiviruses*, known pathogens of some agricultural crops, are multisegmented and have a double-shelled and icosahedral structure [53,55]. The Nilaparvata lugens reovirus (NLRV) has the same structural properties as fijiviruses, but it only replicates in the insect host (rice brown planthopper) [56] whereas other fijiviruses can also replicate in phloem cells of susceptible Gramineae hosts (in which they induce small tumors or enations) or Liliaceae [55]. Previously, most insect-specific fijiviruses have been detected in Hemiptera, which have the potential to be plant virus vectors. However, to the best of our knowledge, this is the first report of a fijivirus from an adult beetle (Coleoptera). The Hubei reo-like virus 1, which has been identified from a Coleopteran host in China, has been categorized as unclassified Riboviria [48] and showed a 23.5% sequence similarity and 68% overlap with our newly identified virus. As most of these recently identified viruses originated from metagenomics studies, there is a possibility for incorrect host assignment, and further studies are necessary to validate the host for these novel viruses.

There are some other members of the *Reoviridae* that infect insects, and they are mainly classified under the genera *Orbivirus* and *Cypovirus*. Orbiviruses, such as the *Umatilla* virus [57], *Aedes* orbi-like virus [58], *Anopheles hinesorum* orbivirus [59], and bluetongue virus [60], are previously reported from different species of mosquitoes and midges across the world, including Australia. *Cypovirus* is another genus in this family that infects insects, such as the Heliothis armigera cypovirus [61] and Bombyx mori cypovirus [62], which are transmitted by contact or faecal–oral routes and, in some cases, cause significant mortality in insect populations [55]. The phylogenetic analysis set this novel canegrub reovirus apart from other orbiviruses and cypoviruses and classified it with other fijiviruses.

### 3.4. Canegrubs’ Sobemo-like Viruses

**Figure 5:**
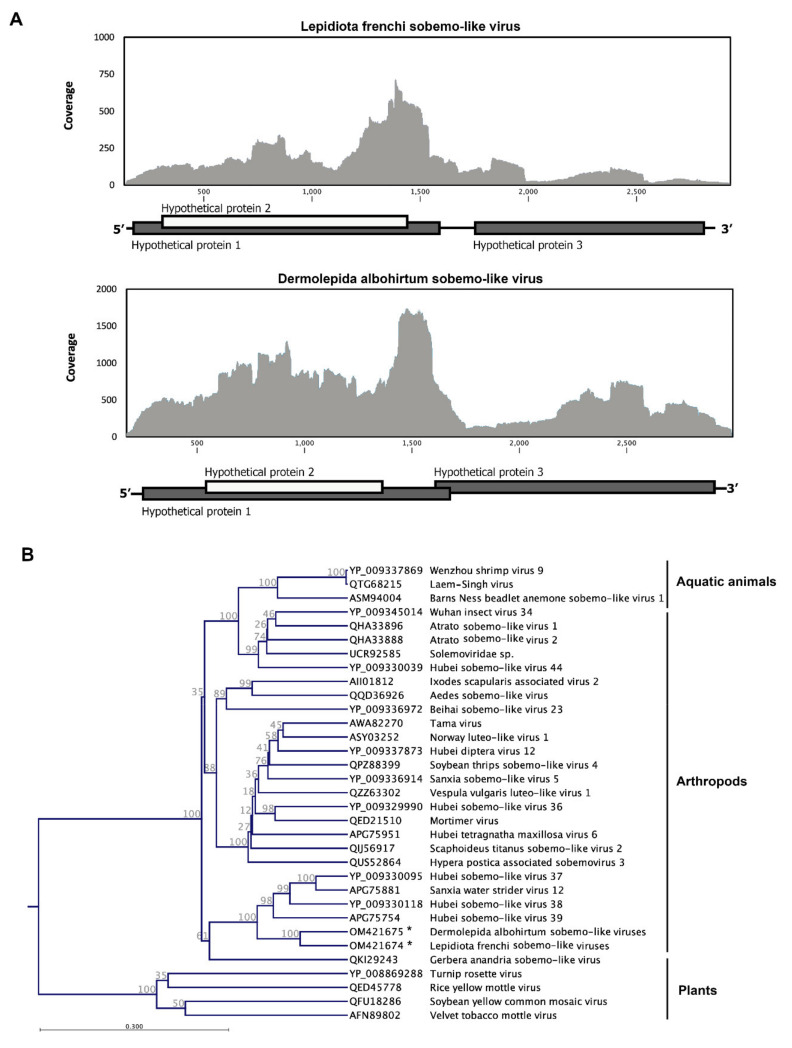
Schematic of the genome organization and coverage of two newly identified sobemo-like viruses from Australian canegrubs. The DaSV genome was built by 11,244 assembled reads that showed an average coverage of 283.01× while the LfSV genome was built by only 2893 assembled reads with an average coverage of 96.86×. RNA-Seq read density plotted along the viral genome. The *Y*-axis shows the number of reads mapped to a particular nucleotide of the virus genome at the *X*-axis (**A**). The maximum likelihood phylogeny of canegrubs sobemo-like viruses (DaSV and LfSV) and previously reported sobemoviruses based on deduced amino acid sequence of ORF3 (conserved RdRP domain). DaSV and LfSv were classified with other unassigned sobemo-like viruses that previously reported from insects and other arthropods. The plant sobemoviruses have been clustered in another clade. The identified virus in this study is shown with an asterisk (**B**).

We identified two sequences in greyback (*D. albohirtum*) and French’s canegrubs (*L. frenchi*) that showed significant similarity to sobemo-like viruses. The Dermolepida albohirtum sobemo-like viruses’ (DaSV) complete genome sequence consists of a single segment of 2761 nucleotides (Figure 5A) with three predicted open reading frames without a poly(A) tail. The genome sequence has 71 and 33 nucleotides in its 5′ and 3′ UTR, respectively, and it is accessible with the NCBI Genebank accession number of OM421675. The Lepidiota frenchi sobemo-like viruses (LfSV) genome sequence consists of 2736 nucleotides and encodes three hypothetical proteins (Genebank accession number: OM421674). In both recently identified sobemo-like viruses, ORF 2 is part of ORF 1, and there is a 119-nucleotide gap between ORF 3 and ORF 2 in LfSV while ORF3 in DaSV has 97 nucleotides overlapped with the first ORF (Figure 5B). A putative RNA-dependent RNA polymerase (RdRP) conserved domain has been identified on ORF 3 while we could not detect any other conserved domains in other ORFs. These two novel viruses showed a 61.9% identity in their entire genome sequence, and a 54.5% similarity has been observed between their deduced amino acid sequence of ORF 3.

Sobemoviruses are groups of plant viruses with linear and nonsegmented positive sense ssRNA genomes, named after their type species, *southern bean mosaic virus* (SBMV) [63] Generally, sobemoviruses are transmitted by insect vectors or by plant seeds [64]. It has been shown that some of these viruses, such as the *rice yellow mottle virus* (RYMV), *southern cowpea mosaic virus* (SCPMV), and *turnip rosette virus* (TRoV), are transmitted by beetles [63]. Some plant viruses can be found in the insect gut cells, and they can enter the hemocoel even through the peritrophic membrane-lined midgut. The southern bean mosaic virus (SBMV) are found in the hemocoel of southern corn rootworm, *Diabrotica undecimpunctata,* after they have fed on an infected plant material [54].

There is no report on the pathogenicity of these viruses in insects. Previous studies proposed that the Laem-Singh virus (LSNV) can be a potential cause of retarded growth syndrome in the giant tiger shrimp, *Penaeus monodon,* in Thailand; however, this virus has been also detected in healthy shrimps in other countries [65]. Recent studies found the genome sequence of several new sobemo-like viruses in a wide range of invertebrate species and classified them to a novel clade that is phylogenetically divergent but related to sobemoviruses [48]. These unclassified sobemo-like viruses have been identified through metagenomic studies in many mosquitos, such as *Aedes albopictus* from southern Switzerland [66], *Ae. vexans nipponii* from the Republic of Korea [67], and *Armigeres subalbatus* from Thailand [68]. They are also among the dominated virome of termites, and five sobemo-like viruses have been reported from various Australian termite species [69]. Therefore, more recent information suggests that sobemo-like viruses are not confined to plants as they have been isolated from many aquatic and terrestrial animals. As several sobemo-like viruses have been found in nonherbivorous or in blood-sucking insects, the food-borne spillover or direct transmission of these viruses from plant materials is very unlikely.

The maximum likelihood phylogeny separated these novel viruses from other plant sobemoviruses, such as the velvet tobacco mottle virus and the soybean yellow common mosaic virus (Figure 5). DaSV and LfSV were classified with other unassigned sobemo-like viruses, which was previously reported from insects and other arthropods. The deduced amino acid sequence of ORF3 of both DaSV and LfSV showed more than 50% similarity and 70% coverage to the Hubei sobemo-like virus 39, which was isolated from mosquitoes in China. Sanxia water strider virus 12 is another closely related virus to DaSV and Lf SV which isolated from water striders in China. Other sobemo-like viruses that isolated from birds (*Dendrocopos leucotos*) and spirurian nematodes are also clustered under the same clades with these novel viruses (Figure 5B). Further studies are necessary to isolate these viruses and determine their infectivity assays in the cell line of canegrubs larvae and adult beetles. At this stage, we do not know about the host specificity of these viruses as well as the possibility of their pathogenicity in other hosts.

### 3.5. Lepidiota Negatoria Beny-like Virus (LnBV)

**Figure 6:**
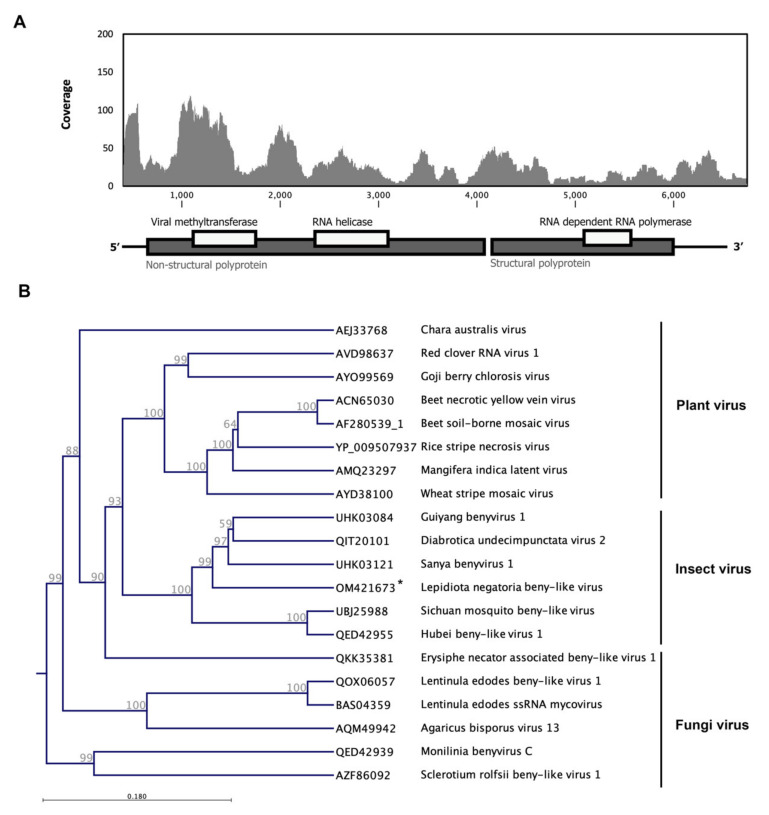
Schematic of the genome organization and coverage of *Lepidiota negatoria* beny-like virus (LnBV). RNA-Seq read density plotted along the viral genome. The *Y*-axis shows the number of reads mapped to a particular nucleotide of the virus genome at *X*-axis. Only 1350 reads mapped to this draft genome sequence (**A**). The maximum likelihood phylogeny classified this novel virus with other insect-specific, beny-like viruses and separated them from plant and fungi viruses. The identified virus in this study is shown with an asterisk (**B**).

From the negatoria canegrub (L. *negatoria*), a 6371-nucleotide (nt) contig was assembled corresponding to the benyvirus and tentatively named Lepidiota negatoria beny-like virus (LnBV). The draft genome of this novel virus contains two open reading frames, one encoding a nonstructural protein, which is located between 146 and 3571 nucleotides (1142 aa) from the 5′ end of the genomic RNA, and another one encoding a structural protein with an RdRp-conserved domain located between 3586 and 5664 nucleotides (693 aa) from the 5′ end of the genomic RNA. Only 1350 reads from the total RNA library mapped to this sequence, and we could not recover this contig from the poly(A)- enriched RNAseq library data (Figure 6A). The annotated genomic sequence of this virus has been deposited in the GenBank under the accession number OM421673.

The molecular weight of the nonstructural protein was estimated at 28.04 kDa, and a conserved protein domain search revealed two conserved viral methyltransferase and RNA helicase domains on this sequence. Through the BLASp search, the deduced amino acid sequence of ORF 1 showed 52% identity and >70% coverage to the nonstructural protein of Du V2, which isolated from the southern corn rootworm, *D. undecimpunctata,* in North America [70] and the Guiyang benyvirus 1, which reported from the Asian lady beetle (*Harmonia axyridis*) in China. The predicted molecular weight of the structural protein with a conserved RdRp domain is 77.95 kDa (isoelectric point of 7.96). The translated open reading frame of the structural protein aligned by 51.2% to RdRp of the Sanya benyvirus 1, which reported from the Asiatic pink stem borer, *Sesamia inferens,* in China (Figure 6).

*Benyvirus* is the only described genus in family *Benyviridae.* This multipartite virus with rod-shaped virions infects plant and transmits by root-infecting vectors. Their single-stranded, positive-sense RNA genome has a 3′ poly(A) tract [71]. Beet necrotic yellow vein virus causes very destructive soil-borne ‘rhizomania’ disease of sugar beet across the globe. However, recent metagenomic studies found some other benyvirus-related sequences in the libraries of a few herbivorous insects, such bark beetles, southern corn rootworms, and also blood-sucking insects such as *Rhodnius prolixu* [70,72].

Phylogenetic analysis shows these benyvirus-related sequences seem to correspond to a distinct species of the currently approved genus, *Benyvirus*. The other unassigned members of this family have infected or currently infect a wide range of hosts, including insects. The maximum likelihood phylogeny classified this novel virus with other insect-specific beny-like viruses and separated them from plant and fungi viruses (Figure 6B).

## 4. Conclusions

Currently, our knowledge of canegrub viruses and their interactions with their hosts is very limited. However, insect-specific, small RNA viruses (SRV) could be important components of pest management in the future. This study provided the complete genome sequences of some novel viruses from canegrubs, but their mode of transmission and pathogenicity are completely unknown. Since these recently identified novel viruses only originated from a metagenomics study, the method would not differentiate sequences from the insect host, its previous ingested meal, or a contaminating parasite or fungus. Incorrect host assignment is the major challenge and limitation of this sort of metagenomic analysis. Future studies are essential to determine the natural incidences of these viruses in their different life stages of the hosts, biodiversity, geographical distributions, and potential as biological control agents for these important pests of sugarcane.

## Figures and Tables

**Table 1:** Previously identified viruses from scarab beetles.

Virus Name	Taxonomy	Host Common Name	Scientific Name	Location	Reference
Poxi-like virus	*Poxviridae*	Cockchafer (maybug)	*Melolontha* spp.	Poland	[11]
Poxi-like virus	*Poxviridae*	Dung beetles	*Geotrupes* spp.	Poland	[12]
Entomopoxvirus	*Poxviridae*	Cockchafer (maybug)	*Melolontha melolontha*	Turkey	[13]
Entomopoxvirus	*Poxviridae*	Cupreous chafer	*Anomala cuprea*	Japan	[14]
Entomopoxvirus	*Poxviridae*	Rose beetle	*Adoretus versutus*	Fiji	[15]
Manawatu virus	*Nodaviridae*	Grass grub	*Costelytra zealandica*	New Zealand	[16]
Flock house virus	*Nodaviridae*	Grass grub	*Costelytra zealandica*	New Zealand	[17,18]
Black beetle virus	*Nodaviridae*	African black beetle	*Heteronychus arator*	New Zealand	[19]
Iridescent virus 6	*Iridoviridae*	June bugs	*Phyllophaga vandinei*	USA	[20]
Small iridescent virus	*Iridoviridae*	Cranberry white grub	*Phyllophaga anxia*	Canada	[21]
Small iridescent virus	*Iridoviridae*	African black beetle	*Heteronychus arator*	South Africa	[22]
Blue iridovirus	*Iridoviridae*	Japanese beetle	*Popillia japonica*	Terceira Island, Portugal	[23]
Small iridescent virus	*Iridoviridae*	Grass grub	*Costelytra zealandica*	New Zealand	[24]
Small iridescent virus	*Iridoviridae*	African black beetle	*Heteronychus arator*	South Africa	[22]
Oryctes rhinoceros nudivirus	*Nudiviridae*	Coconut rhinoceros beetle	*Oryctes rhinoceros*	Asia, Pacific	[25]
Allomyrina dichotoma nudivirus	*Nudiviridae*	Korean horn beetle	*Allomyrina dichotoma*	Korea	[26]
Oryctes rhinoceros picorna-like virus	*Picornavirales*	Coconut rhinoceros beetle	*Oryctes rhinoceros*	Taiwan	[27]
Unknown virus	Unknown	Grass grub	*Costelytra zealandica*	New Zealand	[28]
Spheroidosis viruses	Unknown	Black soil scrab	*Othnonius batesi*	Australia	[29]
Lake Grasmere virus	Unknown	Grass grub	*Costelytra zealandica*	New Zealand	[30]

**Table 2:** Summary statistics of RNA-Seq libraries, mapping to proxy genome references and the de novo assembly.

Samples	Raw Reads	Clean Reads	Genome 1 (*Oryctes rhinoceros*)		Genome 2 (*Onthophagus taurus*) *		Assembly Overview		
			Mapped (%)	Unmapped Reads	Mapped (%)	Unmapped Reads	Contigs >500 bp	N50	L50 (bp)
*Dermolepida albohirtum*									
Total RNA-Seq	90,875,308	90,823,228	73.19%	24,333,795	28.47%	17,405,199	31,911	1373	7905
mRNA-Seq	73,406,632	70,531,633	24.19%	51,654,119	6.64%	48,224,340			
*Lepidiota negatoria*									
Total RNA-Seq	83,850,940	83,784,724	85.17%	12,418,859	13.34%	10,762,711	30,155	1503	7428
mRNA-Seq	75,752,788	72,943,712	18.87%	57,271,175	4.94%	54,442,653			
*Lepidiota frenchi*									
Total RNA-Seq	81,565,214	81,520,706	88.98%	8,978,364	7.45%	8,309,559	26,973	1403	6889
mRNA-Seq	69,835,996	67,249,833	18.95%	52,746,565	5.2%	49,958,261			

* Unmapped reads to first proxy genome reference were used for second mapping.

## Data Availability

Deep sequencing raw data was deposited in the National Centre for Biotechnology Information’s (NCBI’s) Sequence Read Archive (SRA) and are accessible through BioProject series accession number PRJNA798832.
